# Comparative transcriptomic analysis of the super hybrid rice Chaoyouqianhao under salt stress

**DOI:** 10.1186/s12870-022-03586-w

**Published:** 2022-05-07

**Authors:** Guo Xia-Yu, Zhang Meng, Zhu Ming-Dong, Long Ji-Rui, Wei Zhong-Wei, Li Jian-Wu, Zhou Bin, Ai Zhi-Yong, Deng Hua-Feng

**Affiliations:** 1 College of Agronomy, Hunan Agricultural University, Changsha, 410125 P. R. China; 2 National Innovation Center of Saline-Alkali Tolerant Rice in Sanya, Sanya, 572000 P. R. China; 3 Hunan Hybrid Rice Research Center, Changsha, 410125 P. R. China; 4 Hunan Key Laboratory of Plant Functional Genomics and Developmental Regulation, College of Biology, Hunan University, Changsha, 410082 P. R. China; 5 Key Laboratory of Indica Rice Genetics and Breeding in the Middle and Lower Reaches of Yangtze River Valley, Hunan Rice Research Institute, Changsha, 410125 P. R. China; 6 Hunan Academy of Agricultural Sciences, Changsha, 410125 P. R. China

**Keywords:** Super hybrid rice, CY1000, Salt tolerance, Molecular mechanism, Ubiquitination

## Abstract

**Background:**

Soil salinization is a threat to food security. China is rich in saline land resources for potential and current utilization. The cultivation and promotion of salt-tolerant rice varieties can greatly improve the utilization of this saline land. The super hybrid rice Chaoyouqianhao (CY1000) is one of the most salt-tolerant rice varieties and is widely used, but the molecular mechanism underlying its salt tolerance is not clear.

**Results:**

In this study, the characteristics of CY1000 and its parents were evaluated in the field and laboratory. The results showed that aboveground parts of CY1000 were barely influenced by salt stress, while the roots were less affected than those of its parents. A comparative transcriptomic strategy was used to analyze the differences in the response to salt stress among the male and female parents of CY1000 at the seedling stage and the model indica rice 93–11. We found that the salt tolerance of CY1000 was mainly inherited from its male parent R900, and its female parent GX24S showed hardly any salt tolerance. To adapt to salt stress, CY1000 and R900 upregulated the expression of genes associated with soluble component synthesis and cell wall synthesis and other related genes and downregulated the expression of most genes related to growth material acquisition and consumption. In CY1000 and R900, the expression of genes encoding some novel key proteins in the ubiquitination pathway was significantly upregulated. After treatment with MG-132, the salt tolerance of CY1000 and R900 was significantly decreased and was almost the same as that of the wild type after salt stress treatment, indicating that ubiquitination played an important role in the salt tolerance mechanism of CY1000. At the same time, we found that some transcription factors were also involved in the salt stress response, with some transcription factors responding only in hybrid CY1000, suggesting that salt tolerance heterosis might be regulated by transcription factors in rice.

**Conclusion:**

Our results revealed that the ubiquitination pathway is important for salt tolerance in rice, and several novel candidate genes were identified to reveal a novel salt tolerance regulation network. Additionally, our work will help clarify the mechanism of heterosis in rice. Further exploration of the molecular mechanism underlying the salt tolerance of CY1000 can provide a theoretical basis for breeding new salt-tolerant rice varieties.

**Supplementary Information:**

The online version contains supplementary material available at 10.1186/s12870-022-03586-w.

## Introduction

Soil salinization and secondary salinization have become global ecological problems affecting cultivated land and threatening world food security [1]. China’s inland saline-alkali land area is nearly 100 million hectares, and the coastal beach area is nearly 2.333 million hectares [2]. Eighty percent of saline-alkali land is in a state of desolation. In total, 13.33 million hectares of this saline-alkali land is rich in rainwater resources and can be used for rice cultivation in the near future, with great potential for comprehensive utilization. This will be the most economical and effective way to develop and utilize coastal beaches and inland saline-alkali land and guarantee land area for the cultivation and popularization of saline-alkali-tolerant rice varieties [3].

Under the leadership of academician Yuan Longping, the National Hybrid Rice Research Centre has selected a number of salt-tolerant rice varieties with adaptation to salinity levels of 0.3–0.6% and yields of over 4500 kg/ha. Among them, the super hybrid rice variety “Chaoyouqianhao” (CY1000) is the best and is widely used [4]. CY1000, belonging to the fifth generation of super hybrid rice, is an indica two-line hybrid rice variety. It is a cross between the super rice variety R900 and photothermosensitive two-line male sterile line Guangxiang24S (GX24S). CY1000 passed the national examination and approval (national rice examination 20,170,053) in 2017. Although the salt tolerance advantage of CY1000 is being gradually utilized, its molecular mechanism of salt tolerance remains unclear.

To date, studies have found that the main mechanisms of salt resistance are ion balance regulation [5, 6], osmotic balance regulation [7, 8], reactive oxygen species scavenging [9] and nutrient balance regulation [10, 11]. These regulatory effects are mainly mediated by the salt overly sensitive (SOS) pathway and plant hormone signal transduction [12]. When rice is exposed to high salinity, a large amount of Na^+^ enters the cells through univalent cation channels, which stimulates a rapid increase in the intracellular Ca^2+^ concentration [13]. Then, the calcium-binding protein OsSOS3/CBL4 senses this signal, interacts with and phosphorylates OsSOS2/OsCIPK24, a member of the SnRK3 family, and rapidly activates the Na^+^/H^+^ antiporter OsSOS1 on the plasma membrane, which promotes Na^+^ excretion from cells and maintains ion balance in cells [14, 15]. This is the primary mechanism underlying the response of rice to salt stress. Subsequently, plant hormones (such as ABA and ethylene) and epigenetic regulation also respond to salt stress signals and play an important role in plant salt tolerance [11, 16, 17].

To explore the salt tolerance mechanism of CY1000, we used comparative transcriptomics to examine the response of CY1000 to salt stress at the seedling stage, analyzed the differences in gene expression among male parents, female parents and the model indica rice Line 93–11, and analyzed the molecular mechanism of CY1000 salt tolerance to provide a theoretical basis for breeding new salt-tolerant rice varieties.

## Results

### The field planting results of CY1000 showed that it had good salt tolerance and high yield in high-salt fields

**Fig. 1:**
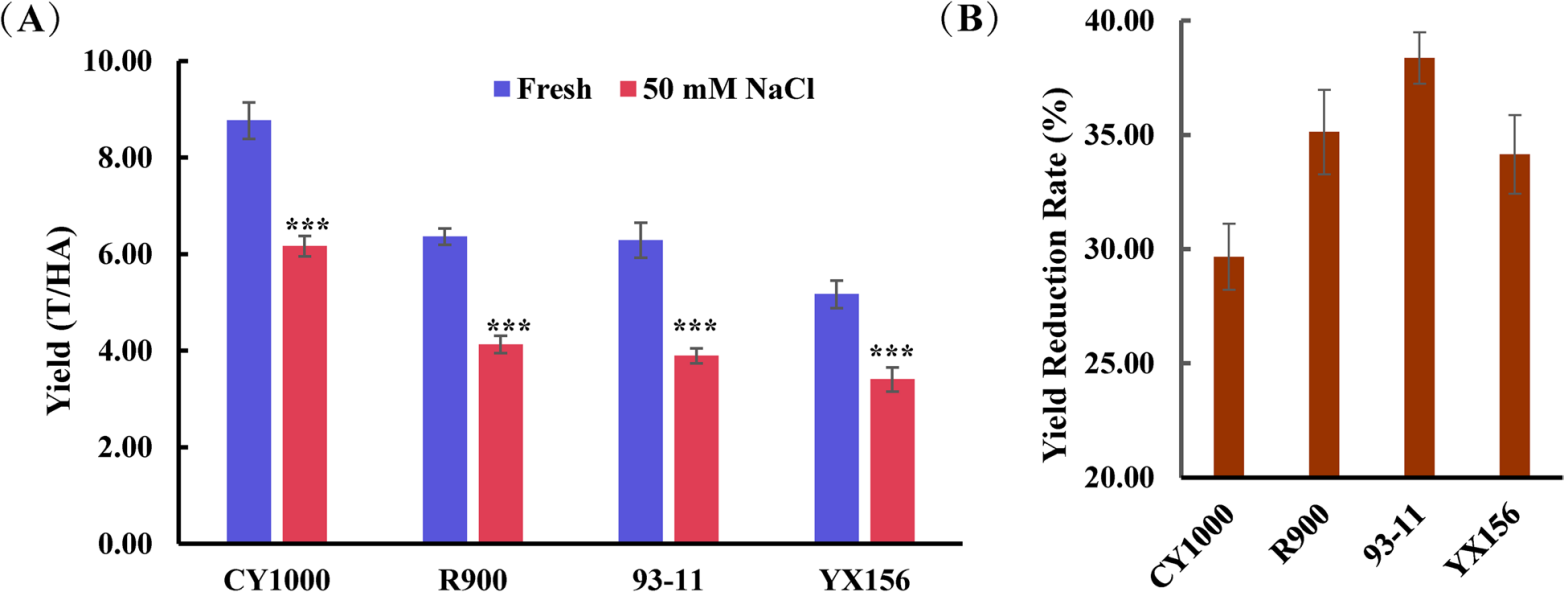
Yield performance of CY1000 under salt stress. **A** The yield of different varieties under 0.3% salt stress. **B** Salt stress (0.3%) reduced the yield of different varieties

From 2018 to 2020, field experiments were carried out in Sanya city, Hainan Province (109.17°E, 18.36°N), for three consecutive years (Fig. 1). CY1000 exhibited advantages in seed setting rate, yield under 0.3% salt stress and yield reduction under 0.3% salt stress (Fig. 1B). In 2020, 13.3 ha of CY1000 was planted and subjected to 0.3% salinity, and the average yield was 7.6 T/ha (Fig. 1A).

### CY1000 and its male parent R900 had high salt tolerance, but its female parent GX24S showed almost no salt tolerance

**Fig. 2:**
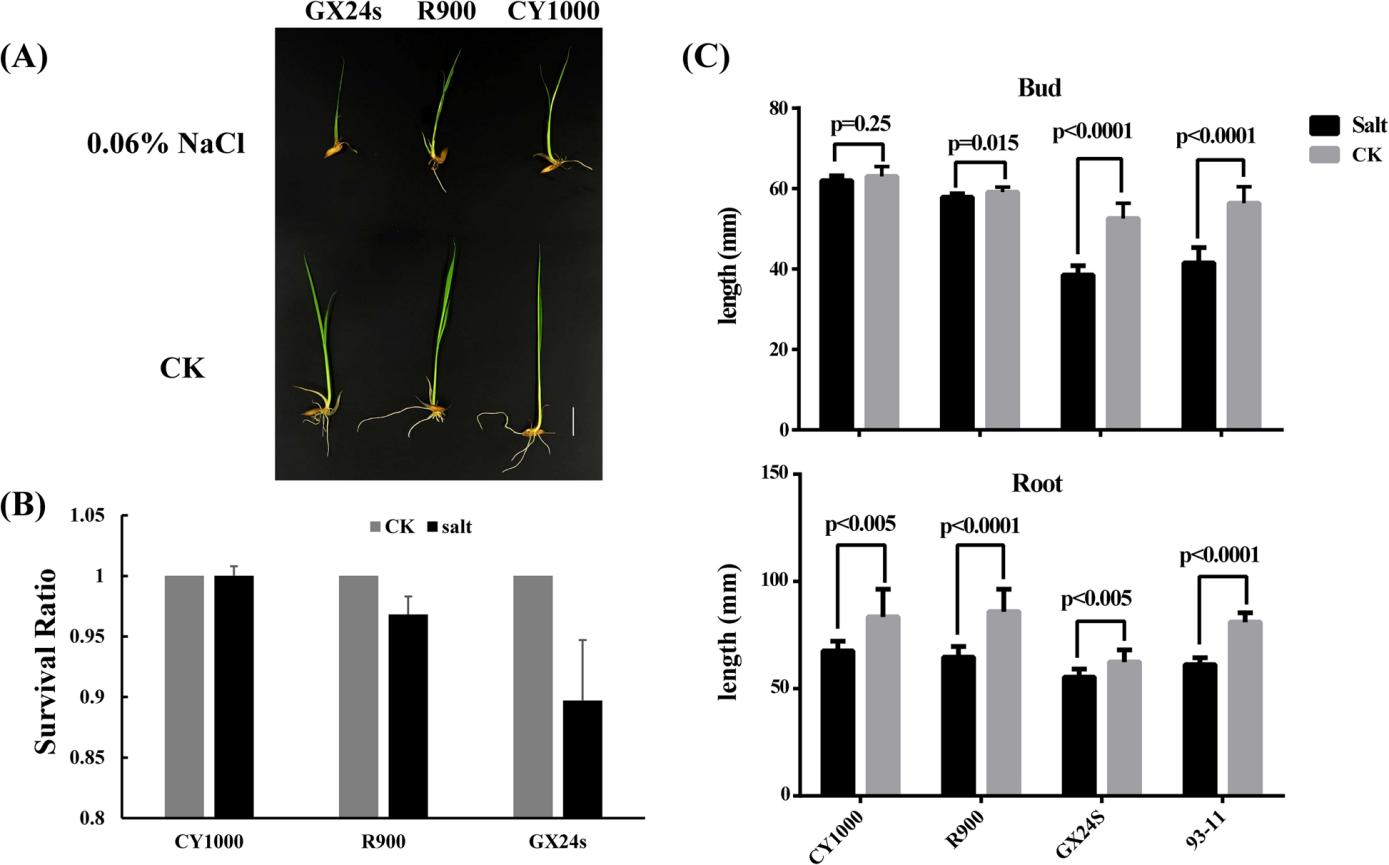
Results of salt tolerance treatment of seedlings of CY1000 and its parents. **A** Photos of 7 d salt-treated and control rice seedlings (scale bar, 1 cm). **B** Survival rate of CY1000 and its parents after 7 d of salt treatment. **C** The length of roots and stems of three rice seedlings after 7 d of salt treatment. Each value was verified by more than 20 samples. The *p* value was tested by Student’s t test

To further understand the salt resistance mechanism of CY1000, the responses of the parents and the model species 93–11 to salt stress at the seedling stage were analyzed. When 7-day-old seedlings of CY1000, the parents R900 and GX24S, and 93–11 were planted in 0.6% NaCl, the growth of the four varieties was inhibited to varying degrees after 7 d. CY1000 showed the strongest salt tolerance (Fig. 2A-B); the survival rate was still 98% after 7 d, and the stem length was barely different from that of untreated CY1000 (Fig. 2B-C). The growth of the female parent GX24S and that of 93–11 was significantly inhibited; the survival rate decreased to below 40%, and the growth of the stems and roots was significantly affected, showing a state of stopped growth. In addition, the male parent R900 also showed strong salt tolerance; the survival rate reached 85%, and the length of the stem showed no significant difference compared to that of the untreated materials (Fig. 2B&D). Interestingly, the roots of CY1000 and R900 were also influenced by salt stress, but they also exhibited no growth inhibition. After careful observation, it was found that there were obvious salt crystals at the stem nodes of CY1000 seedlings, which indicates that the balance of the Na^+^ emission concentration is one of the important factors underlying salt resistance.

### Comparative omics revealed the molecular mechanism by which CY1000 and its parents respond to salt stress

**Fig. 3:**
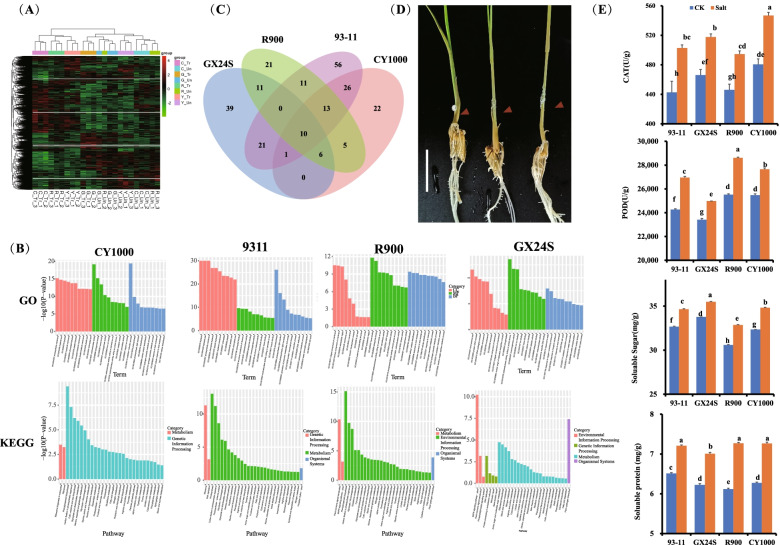
Differences in CY1000 and other varieties under salt treatment. **A** Heatmap of all expressed genes from samples. **B** GO and KEGG functional analyses of the three varieties under salt treatment. **C** Venn diagram of GO terms among samples. **D** Observation of the salt tolerance and phenotype of CY1000. Salt crystals were observed in the nodes of CY1000 stems

In total, 6.3 Gb of data was obtained for each replicate of each sample, with Q20 over 95.8% and Q30 over 92%. The total amount of data was more than 163 Gb. More than 106.8 Gb of clean reads was obtained. When using parametric analysis, the proportion of the total mapped genes in the genome was more than 91% in most samples; only the proportion of R_Un_1 was 83.66%. Further statistics on the distribution of reads on the genome showed that 88.45% of the reads were mapped to genes, and 96.27% of the reads were mapped to exons. The PCA and Pearson correlation results are shown in Fig. S1. Although a few replicates were not most correlated, they all clustered separately. RNA sequencing (RNA-Seq) showed that the number of differentially expressed genes in 93–11 and GX24S was quite different (Fig. S1, Table S1). GX24S had the fewest (2940 DEGs, including 1685 downregulated and 1255 upregulated genes) differentially expressed genes, while 93–11 had the highest number (5809 DEGs, including 2793 downregulated and 3016 upregulated genes) of differentially expressed genes; CY1000 had 5261 DEGs, including 2828 downregulated and 2433 upregulated genes; and R900 had 4114 DEGs, including 2292 downregulated and 1822 upregulated genes (Fig. 3A, Fig. S1B-E).

After clustering the expression patterns, all samples were divided into two branches according to treatment and no treatment, indicating that there were common mitigation patterns in the salt stress response among different varieties at different levels (Tables S2, S3, S4 and S5). The response patterns of GX24S were the most similar, indicating that they were insensitive to salt stress, which may be the reason for the poor salt resistance. CY1000 and R900 had more similar expression spectra, which indicates that the salt resistance of CY1000 was inherited from R900.

**Fig. 4:**
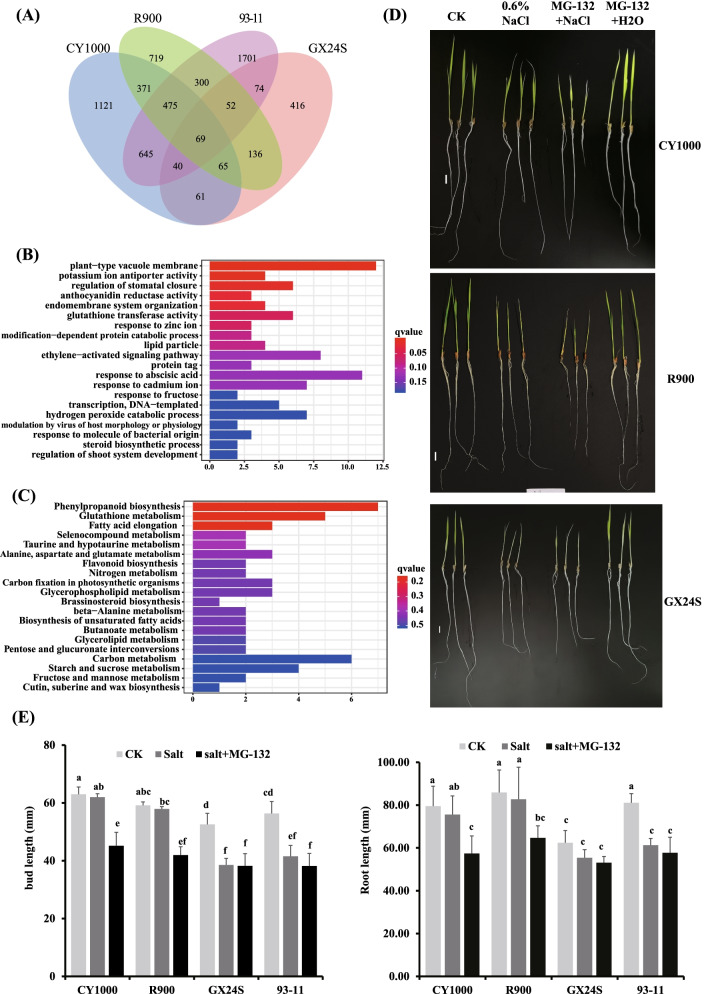
Ubiquitination is the main mechanism underlying the salt tolerance of CY1000. **A** Venn diagram of DEGs among different rice varieties. **B-C** The GO and KEGG functional annotation of 371 DEGs specifically expressed in CY1000 and R900. D. Photos of rice seedlings under salt stress after MG-132 treatment

GO and KEGG functional enrichment analyses also showed that CY1000 and R900 had more similar response patterns, including “phenylpropanoid biosynthesis”, “carbon fixation in photosynthetic organization”, “response to oxidation” and other pathways, which were not enriched in 93–11 and showed no difference in GX24S, indicating that they play a certain role in salt tolerance (Figs. 3B-C, 4B).

After clustering the upregulated and downregulated genes, it was found that for salt tolerance, the upregulated genes of CY1000 and R900 were involved in oxidative stress, soluble component synthesis, and cell wall synthesis, while the downregulated genes were involved in the acquisition and consumption of most growth substances (Tables S2 and S3). The above results indicate that oxidative stress, cell wall synthesis, soluble sugars and other metabolic pathways also play a role in the salt stress resistance of CY1000 and R900, which is consistent with other research results [18–21]. Then, physiological indices, including CAT, POD and soluble sugar and protein, were detected (Fig. 3E). According to the results, after salt treatment, all four phenotypes showed increases in these activities, but CY1000 and R900 had higher rates of increase than 93–11 and GX24S, suggesting that these two salt-tolerant phenotypes had more sensitive response mechanisms with regard to these activities.

Interestingly, GO and KEGG functional enrichment analyses showed that CY1000 was most similar to 93–11, followed by R900, and most of the functional modules that differed were the same in the two comparisons, but there was only one overlap with GX24S, indicating that the salt resistance of CY1000 was inherited from R900 but had some “atavism”, which may be part of the reason for heterosis (Fig. 3B).

### Ion balance and ubiquitination are key factors affecting the salt tolerance of CY1000

To further reveal the unique salt resistance mechanism of CY1000 and identify the most critical regulatory nodes, we analyzed the four varieties by Venn diagram analysis. A total of 371 genes were specific to CY1000 and R900, which means that these genes are the most critical genes for salt resistance (Fig. 4A, Table S6). Through enrichment analysis of these 371 genes, it was found that these genes were still enriched in pathways associated with ion concentration regulation (ion response and transport, mainly MFS domain-containing proteins), redox activity (flavone and glutathione metabolism), soluble substances and so on.

**Fig. 5:**
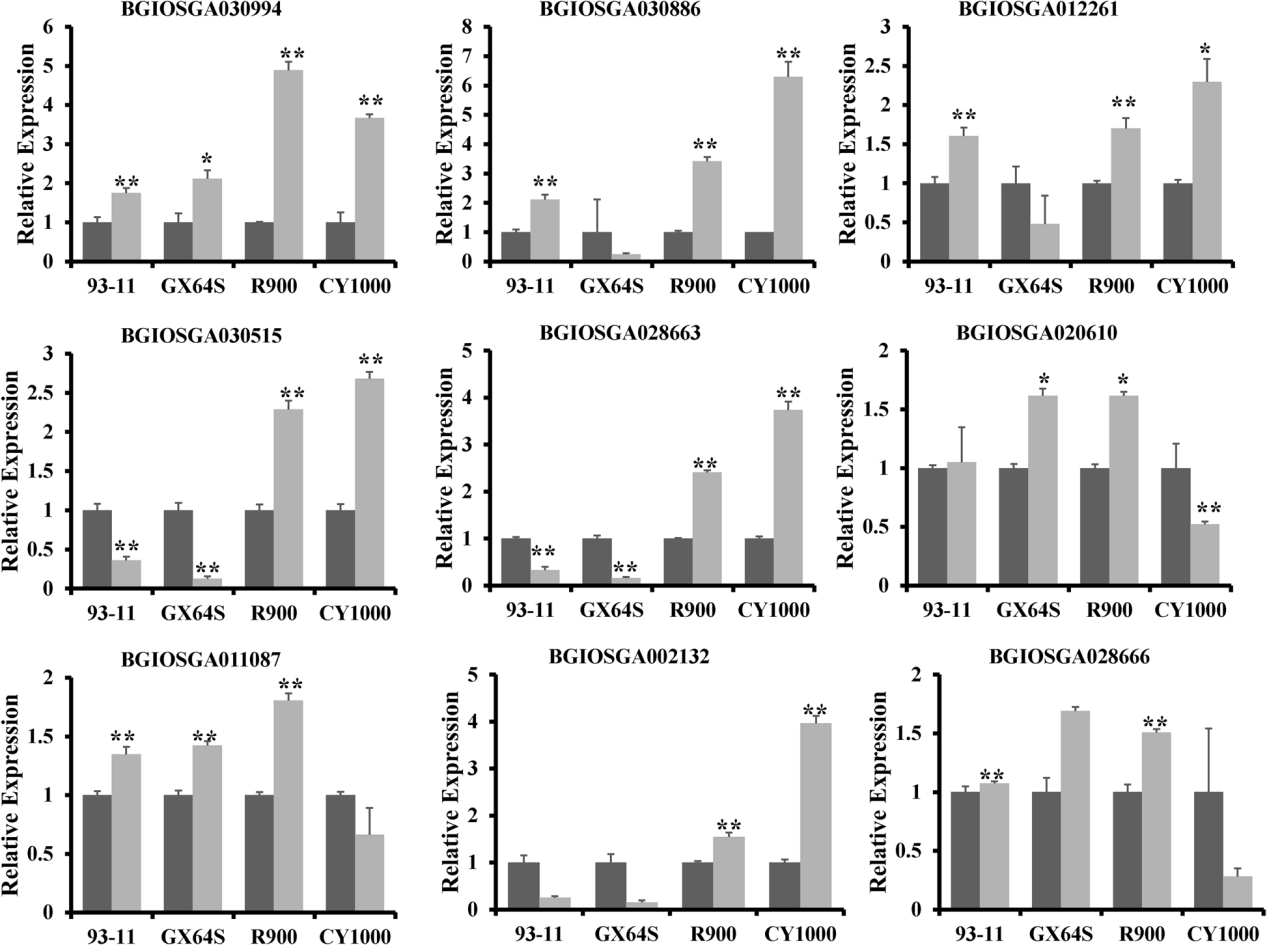
qRT-PCR of selected Ubiquitination genes in treated four phenotypes. Each samples has three bio-replicates, and it was tested by student’s t-test. * represents *p*-value < 0.05 and ** represents *p*-value< 0.01

Among the downregulated genes, those encoding MFS domain-containing proteins, protein kinase domain-containing proteins and peroxidases were the most important. Among the upregulated genes, those encoding ubiquitin-like domain-containing proteins (Ubxs) and F-box domain-containing proteins, which were all previously unidentified, accounted for the majority; this indicates that enhanced ubiquitination is another reason for the strong salt resistance of CY1000 and R900 (Table 1, Fig. 5, Table S6).

**Table 1:** Salt-responsive ubiquitination genes in CY1000 and R900

	Gene	log2FC (CY1000)	padj	log2FC(R900)	padj	log2FC(GX24S)	padj	log2FC(93–11)	padj
Ubiquitin-like domain-containing protein	BGIOSGA030994	6.84	0.01	5.96	0.00	−0.09	1.00	2.90	0.40
	BGIOSGA030886	7.17	0.00	6.17	0.00	−0.95	1.00	6.14	0.27
	BGIOSGA012261	4.06	0.00	3.09	0.01	−0.96	1.00	1.97	0.28
F-box domain-containing protein	BGIOSGA030515	5.81	0.00	5.67	0.02	−0.17	1.00	Inf	0.11
RING-type domain-containing protein	BGIOSGA028663	2.61	0.00	1.87	0.05	−0.03	1.00	2.66	0.09
	BGIOSGA020610	−1.94	0.00	−1.64	0.01	−0.90	0.38	−1.05	0.08
	BGIOSGA011087	−1.24	0.03	−1.32	0.02	−0.59	0.85	−1.03	0.09
	BGIOSGA006792	2.32	0.01	2.24	0.02	0.91	1.00	3.11	0.16
	BGIOSGA002132	2.85	0.00	2.52	0.00	0.03	1.00	0.98	0.54
RING-type E3 ubiquitin transferase	BGIOSGA028666	−1.75	0.00	−1.21	0.03	−0.56	1.00	−2.51	0.48

Three Ubx proteins and one F-box domain-containing protein were significantly upregulated (143.9-, 114.3-, 16.6- and 56.1-fold, respectively) after salt stress according to transcriptome data. Additionally, in the qRT–PCR results, these four genes showed a high increase in CY1000, as they had a significant increase in R900. In addition, six ring-type domain-containing proteins were also significantly induced by salt stress (three of them were upregulated more than 5-fold, and the other three were downregulated more than 2-fold), indicating that ubiquitination played an important role in the salt tolerance mechanism of CY1000. Interestingly, according to qRT–PCR, two ring-type domain-containing protein genes, BGIOSGA020610 and BGIOSGA011087, did not show the same decreasing pattern in R900 as in CY1000, so the two genes need to be further functionally studied to reveal their significance in the salt tolerance response.

Then, after the seedlings of CY1000 and its parents were treated with the ubiquitin inhibitor MG-132, the salt tolerance of CY1000 and R900 decreased significantly, and there was almost no difference between the phenotypes of CY1000 and R900 after salt stress treatment (Fig. 4D&E). These results indicate that ubiquitination-mediated regulation is the key pathway of salt tolerance in CY1000.

### Role of transcription factors in the salt tolerance of CY1000

Transcription factors are the main regulatory factors. Among 371 salt resistance genes, 24 transcription factors were found, including 3 ERFs, 3 NACs, 5 bHLHs, and 2 WRKYs (Table S8). Four were upregulated, while 20 were downregulated, indicating that CY1000 and R900 inhibit the acquisition and metabolism of growth substances by downregulating the expression of these transcription factors. Among them, BGIOSGA012597 (ERF) and BGIOSGA018654 (NAC) had higher expression abundance in CY1000, and the difference factor after salt treatment was similar to that observed for R900, but the abundance increased by more than 2.5–3-fold. This finding indicated that this ERF may have a more active regulatory role in the hybrid CY1000, which may explain why CY1000 is more salt resistant. The abundances of BGIOSGA0209257 (NAC), BGIOSGA02290 (B3), BGIOSGA23706 (WRKY) and BGIOSGA02706 (NAC) were low, and these transcription factors were more strongly downregulated after salt treatment. Except for BGIOSGA02090 (B3), the expression abundance of these transcription factors in GX24S was similar to that in R900, indicating that the local expression level and salt stress response of these genes developed after the formation of hybrids.

### Analysis of 1121 differentially expressed genes specific to CY1000 revealed heterosis

We next analyzed the specific salt stress response genes of CY1000 and found that they were mainly enriched in ribosome, C/N metabolism, chlorophyll synthesis, etc., wherein ribosome and other pathways were significantly upregulated, while chlorophyll and other pathways were significantly downregulated. These results indicate that CY1000 not only inherited the salt tolerance mechanisms of R900 but also developed further enhanced salt tolerance characteristics, that is, it underwent heterosis by regulating these mechanisms.

To explore the mechanism underlying the heterosis of CY1000, we studied the transcription factors among the 1121 genes (Table S7). There were 68 transcription factors, including 10 bHLHs, 6 ERFs, 7 NACs, 5 bZIPs and 5 WRKYs. Thirty-two of them were downregulated, and 36 were upregulated. Among them, 6 NACs, 6 bHLHs, 4 ERFs and 4 WRKYs were downregulated, and 4 bZIPs were upregulated. In particular, bHLH BGIOSGA027780 was upregulated 65.5-fold under salt treatment in CY1000. Additionally, three WRKYs (BGIOSGA014175, BGIOSGA036756 and BGIOSGA034613) were downregulated at least 20-fold. One NF-YA (BGIOSGA005501) and two bHLH (BGIOSGA000097 and BGIOSGA006236) were downregulated approximately 10-fold. All these specific genes in hybrids need to be further studied to clarify their relationships with salt tolerance and heterosis.

## Discussion

### The salt resistance of CY1000 hybrids was mainly inherited from R900, and oxidative stress, balance of ion concentration and increase in soluble matter were the reasons for salt resistance

It has been widely reported that the basic model of rice salt resistance involves oxidative stress, ion concentration balance, an increase in soluble matter increases and so on [10–12]. In our results and reported studies, the 93–11 line also has such a response mechanism, but it did not show the corresponding salt resistance, which indicates that in addition to these, it has a stronger salt resistance mechanism (Fig. 3) [22]. In addition, R900 showed the same pattern, and GX24S did not respond to salt stress. This also indicated that the salt resistance of CY1000 was inherited from R900, but CY1000 showed stronger salt resistance (Fig. 2B), reflecting a certain degree of heterosis. However, GX24S had no salt tolerance, which indicated that the heterosis was not caused by superposition. Through multiomic comparative analysis, 371 genes were found to be specific to CY1000 and R900, which indicated that these genes were the main functional genes for salt resistance in the two varieties. These genes were mainly involved in the balance of ion concentration (ion transport, preservation, etc.) and oxidative stress, and included, for example, genes encoding MFS proteins and those involved in the synthesis of antioxidant substances, indicating that these components constitute the main pathways of salt resistance (Fig. 4B&C, Table S6).

### Increased ubiquitination is another important reason for salt resistance

Moreover, we found that Ubx proteins (sharing 100% similarity with ubiquitin; accession numbers a2z1x7, b8bcu7 and a2xeq8) and many F-box domain-containing proteins, including 10 unidentified proteins, were significantly different among the 371 genes, which shows that ubiquitination plays a key role in this process. Previous studies have shown that ubiquitination plays an important role in salt tolerance in plants [23–27]. For example, expression of the *ospub15* gene of rice (*Oryza sativa*) was induced under salt stress, and the root and stem development of the *ospub15* mutant was slow [28]. Moreover, the RNA interference (RNAi)-generated *ospub15* mutant plant was extremely sensitive to salt stress. Further studies have shown that ospub15 can reduce reactive oxygen species (ROS) bursts and cell mortality, thus positively regulating the salt stress response [28]. For example, in *A. thaliana*, the E2-regulated enzymes ubiquitin carrier proteins (UBCs) 1 and 2 regulate the ubiquitination of H2B and then regulate MYB42 by inducing mpk4 expression. This leads to enhanced phosphorylation of MYB42 under salt stress to maintain the stability of MYB42, followed by regulation of the tolerance of plants to salt stress [29].

According to our results, ubiquitination was increased (16-fold for one and more than 114-fold for the other two in CY1000 and more than 8-fold and 62-fold in R900), indicating that ubiquitination plays an important role in the salt resistance of CY1000 and R900. We used MG-132, an inhibitor of ubiquitin degradation, to treat CY1000 and R900 and then observed the changes under salt stress. The salt tolerance of CY1000 and R900 decreased, indicating that ubiquitination played an important role.

In addition, we found that ubiquitination-related genes in CY1000 had a stronger response pattern than those in R900, which may be one of the reasons for the heterosis of CY1000 (Table 1, Table S6).

### CY1000 heterosis occurs due to its more active regulatory mode, in which some transcription factors play a role

According to the above results, we know that the salt resistance of CY1000 was basically derived from R900. However, the difference in the salt resistance phenotype should be due to differences in expression levels. Our transcriptome data also showed that the difference in the expression of 371 genes with common salt resistance was greater in CY1000 (Fig. 4, Table S6). Particularly, among the 371 genes, a series of TFs and functional genes were expressed at higher levels in the hybrid CY1000 than in the parental line R900. For example, two novel TFs, BGIOSGA012597 (ERF) and BGIOSGA018654 (NAC), were 2.5 to 3 times abundant in the hybrid. BGIOSGA018654 was most similar to AtNAC075 (also called SND4), which is considered involved in the negative regulation of flowering and secondary cell wall biosynthesis [30]. BGIOSGA012597 (ERF) was closest to *Arabidopsis* RAP2–3, which is comprehensively involved in stress responses, such as heat, H2O2 and osmotic stresses [31]. According to these results, it can be concluded that these TFs might be valuable for alleviating salt stress, and CY1000 showed better salt tolerance than R900. The conditions for the high expression of these TFs and genes in heteroalacles need to be further clarified to explain heterosis.

Moreover, there were 1121 DEGs specifically expressed in CY1000 but not in R900 (Fig. 4A, Table S7). More responsive genes are a main cause of heterosis, which has already been confirmed in many reports, revealing a more active regulatory mode [22].

Transcription factors are the main regulators in plants, probably the main reason for the active regulation mode in CY1000. In our data, we found that 24 transcription factors may play an important role in the salt tolerance response, and 5 of them showed a relatively strong response, which may play a role in heterosis (Table S8). However, most of the transcription factors did not show obvious expression and response advantages. However, 68 of the 1121 differentially expressed genes specific to CY1000 were not differentially expressed in the parents, indicating that this transcription regulatory network did play an important role in the more active regulation of heterosis. Most of these transcription factor families, including the bHLH, WRKY and NAC families, are involved in salt stress resistance, and some of them have been proven to be salt stress-related factors, such as BGIOSGA011061, which is most similar to DREB26 in *Arabidopsis thaliana* and is related to salt stress [32]. BGIOSGA036921 (NAC domain-containing protein 77, onac132, onac300) also responded to salt stress [33]. Some transcription factors only play a role in the response of CY1000 to salt stress, indicating that the regulation of these transcription factors may be the mechanism of heterosis. Moreover, not only stress-responsive TFs but also many developmental TFs were expressed specifically and extremely differentially in salt-treated CY1000. For instance, BGIOSGA027780 (bHLH), which is similar to HEC3, which is responsible for transmitting tract and stigma development in A*rabidopsis*, was upregulated 65-fold [34]. These results indicated that heterosis was caused by the change in the degree of regulation of some genes after crossing between the two parents, resulting in a more sensitive response to stress.

## Conclusions

Soil salinization is a threat to food security, and China is rich in saline land resources for potential and current utilization. The super hybrid rice variety CY1000 is one of the best salt-tolerant rice varieties and is widely used. Therefore, the analysis of the molecular mechanism of salt tolerance of CY1000 can provide a theoretical basis for breeding new salt-tolerant rice varieties. Therefore, an analysis of a comparative transcriptomic dataset of seedlings of CY1000 and its parental lines R900 and GX24S along with 93–11 under salinity stress and controlled conditions was conducted. CY1000 inherited its salt tolerance from the R900 parent. A total of 371 DEGs were found to be specific to only the salt tolerant phenotypes CY1000 and R900, suggesting that these genes are associated with the salt response. Among these genes, MFS, especially ubiquitination-related genes, exhibited the most responsive patterns. MG-132 pretreatment repressed the salt tolerance of CY1000 and R900, suggesting that increased ubiquitination is important for salt tolerance. Additionally, some candidate ubiquitination-related genes and TFs were found and considered important for the salt response. Additionally, CY1000 also showed a better salt tolerance than the parent R900, indicating that heterosis exists in CY1000. Among the 371 genes, a series of TFs and genes were expressed at higher levels in CY1000 than in R900, supporting the occurrence of heterosis. Moreover, a total of 1121 DEGs specific to CY1000 were found, which could be the other reason for heterosis. Concludingly, our results provide a picture of how CY1000 responds to salt stress and heterosis.

## Materials and methods

### Plant materials, seed germination and salt stress treatment

CY1000 is a super hybrid rice variety cultivated by the Hunan Hybrid Rice Research Center and approved by the state in 2017, and its parents are GX24S and R900; the rice line 93–11 was used as a control. Variety information can be queried at the China Rice Data Center (https://www.ricedata.cn/variety/varis/616642.htm). All rice seeds came from long-term reproduction in our laboratory, and there are no intellectual property disputes related to these materials. The seeds used in this study were harvested in the autumn of 2020, and batches of fresh seeds were placed on wet filter paper for germination, followed by transplantation into seedling pots for hydroponic seedling cultivation for 7 d. Then, some of the seedlings were hydroponically grown in 0.6% (100 mM) NaCl solution, and some of the seedlings were transferred to fresh water solution for culture. After 7 d of growth, the phenotypes were observed, and whole plants were taken for omics sequencing. Each phenotype had three replicates for RNA-seq.

### Total RNA extraction, mRNA purification and cDNA library construction

Total RNA was extracted by TRIzol [35], the gel was removed, the absorbance value was measured, and the quality was detected by an Agilent 2100 Bioanalyzer.

The mRNA containing the poly-A structure was enriched by magnetic beads, and the fragment length was 200–300 bp. Then, the cDNA library (380 bp) was constructed by using 6-base random primers.

### High-throughput RNA sequencing and analysis

Based on the Illumina sequencing platform, these libraries were sequenced by paired-end (PE) (2 * 150 bp) sequencing.

First, the raw data were filtered, and cutadapt [36] was used to remove the sequences with 3′ end connectors. Reads with average mass fractions lower than Q20 were removed. The filtered clean data were aligned with HISAT2 (http://ccb.jhu.edu/software/hisat2/index.shtml) [37]. This software compares filtered reads with a reference genome. HISAT2 uses an improved BWT algorithm. The reference genome in Ensembl was Oryza_ indica.ASM465v1.dna.toplevel.fa.

We used the union scheme of htseq to compare the read count value of each gene, which was used as the original gene expression level. The counts of reads were positively correlated with the real expression levels of genes and the length and sequencing depth of genes. To make the gene expression levels of different genes and different samples comparable, we used fragments per kilobase per million fragments mapped (FPKM) values to standardize the expression levels. FPKM values indicate the number of fragments from a gene per thousand base length per million fragments. For PE sequencing, each fragment has two reads, and the FPKM value is calculated based on only the number of fragments of two reads that can be compared to the same transcript. In the transcriptome, we generally considered genes with FPKM > 1 to be expressed.

### Identification of differentially expressed genes

We used DESeq [38] to analyze the differences in gene expression and used the conditions for differential gene expression as follows: expression difference multiple | log2fold change | > 1, *p* value < 0.05.

### Enrichment analysis

topGO was used for GO enrichment analysis. During the analysis, the gene list and gene number of each term were calculated by using the different genes annotated by GO terms. Then, the *p* value (*p* value < 0.05) was calculated by the hypergeometric distribution method to determine the GO terms with significant enrichment of different genes compared with the whole-genome background to determine the main biological functions of different genes.

### MG-132 treatment

After germination and seedling development, the seeds were treated with 10 μM MG-132 for 6 h before salt treatment and then transferred to salt water for 7 d. The phenotype was observed, and the survival rate was analyzed by ANOVA.

### Physiological index detection and qRT–PCR

Four phenotypes, 93–11, GX64S, R900 and CY1000, were treated with 0.6% NaCl solution, and the whole plant was sampled to detect the CAT and POD activities and the contents of soluble sugar and soluble protein. Additionally, the expression levels of the ten ubiquitination-related genes were tested by qRT–PCR. Actin was used as the reference gene, and all the primers are listed in Table S9. All experiments were conducted with three replicates.

## Supplementary Information

**Additional file 1: Figure S1.** Information on transcriptomic data. A. PCA of all samples. B-E. Gene expression levels of CY1000, R900, GX24S and 93–11. F. Correlation analysis of all samples.

**Additional file 2: Table S1.** Expression levels of all identified genes in all samples. C, R, G and Y are short for CY1000, R900, GX24S and 93–11.

**Additional file 3: Table S2.** DEGs of CY1000 under salt stress treatment.

**Additional file 4: Table S3.** DEGs of R900 under salt stress treatment.

**Additional file 5: Table S4.** T DEGs of GX24S under salt stress treatment.

**Additional file 6: Table S5.** T DEGs of 93–11 under salt stress treatment.

**Additional file 7: Table S6.** The 371 DEGs specifically associated with the salt response.

**Additional file 8: Table S7.** The 1121 DEGs specific to CY1000.

**Additional file 9: Table S8.** 24 TFs specifically responded to salt stress in CY1000 and R900.

**Additional file 10: Table S9.** Primers for the qRT-PCR.

## Data Availability

The raw reads produced in this study were deposited in the NCBI SRA under bioProject number PRJNA785275. The clean data were submitted to SRA under SRP349079.
