# Supplementary figures and images for: Comparative transcriptomic analysis of the super hybrid rice Chaoyouqianhao under salt stress
Source: BMC Plant Biol. 2022 May 7;22:233. doi: 10.1186/s12870-022-03586-w (PMC9077912; doi:10.1186/s12870-022-03586-w)

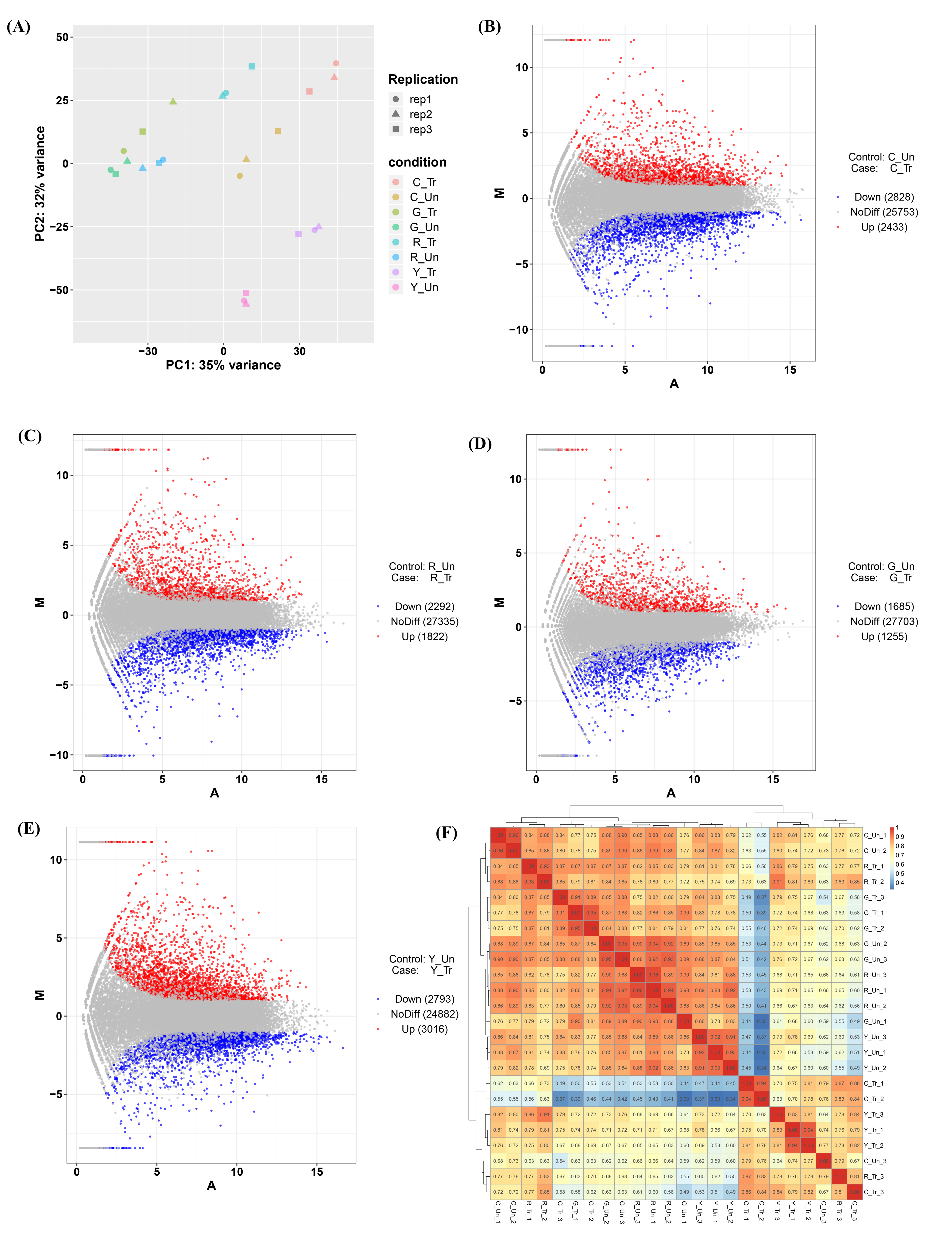

Supplement: Supplementary file 1 — Additional file 1: Figure S1. Information on transcriptomic data. A. PCA of all samples. B-E. Gene expression levels of CY1000, R900, GX24S and 93–11. F. Correlation analysis of all samples. [file 12870_2022_3586_MOESM1_ESM.tif]
